# Supplementary material for: Development and evaluation of the ARM algorithm: A novel approach to quantify musculoskeletal disorder risk factors in manual wheelchair users in the real world
Source: PLoS One. 2024 Apr 2;19(4):e0300318. doi: 10.1371/journal.pone.0300318 (PMC10986926; doi:10.1371/journal.pone.0300318)
Supplement: S1 Appendix — (DOCX) [file pone.0300318.s002.docx]

**Appendix A**

Table 1. Participant characteristics in free-living environment data collection (N= 16)

| **Sex, N(%)** | |
| --- | --- |
| Female | 2 (12%) |
| Male | 14 (88%) |
| **Age, years** | |
| Mean (SD) | 41 (12) |
| Median (IQR) | 37 (31, 55) |
| **Duration of wheelchair use, years** | |
| Mean (SD) | 11 (12) |
| Median (IQR) | 6 (4, 12) |
| **Injury level, N(%)** | |
| C6-C8 | 3 (19%) |
| T1-T8 | 8 (50%) |
| T9-L1 | 5 (31%) |
| **AIS Score, N(%)** | |
| A | 9 (56%) |
| B | 3 (19%) |
| Unknown | 4 (25%) |
| **Self-reported Completeness of SCI, N(%)** | |
| Complete | 8 (50%) |
| Incomplete | 8 (50%) |
