# Supplementary material for: Development and evaluation of the ARM algorithm: A novel approach to quantify musculoskeletal disorder risk factors in manual wheelchair users in the real world
Source: PLoS One. 2024 Apr 2;19(4):e0300318. doi: 10.1371/journal.pone.0300318 (PMC10986926; doi:10.1371/journal.pone.0300318)
Supplement: S2 Appendix — Examples of experimental set-ups for community data collection (a & b): a) wheelchair propulsion in the community and b) car-to-wheelchair transfer with IMU sensors on upper arms. (DOCX) [file pone.0300318.s003.docx]

**Appendix B**


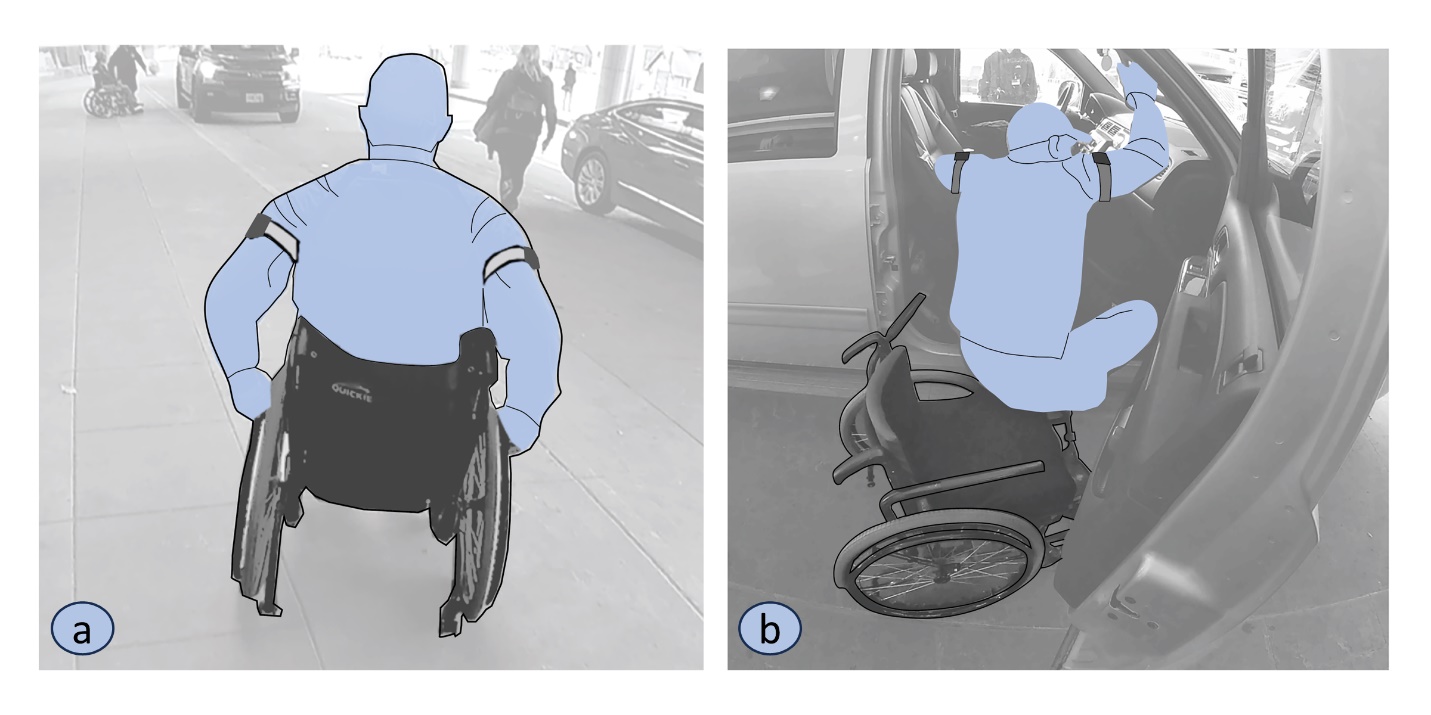


**Figure 1. Experimental Set-up.** Examples of experimental set-ups for community data collection (a & b): a) wheelchair propulsion in the community and b) car-to-wheelchair transfer with IMU sensors on upper arms.
