# Supplementary material for: Development and evaluation of the ARM algorithm: A novel approach to quantify musculoskeletal disorder risk factors in manual wheelchair users in the real world
Source: PLoS One. 2024 Apr 2;19(4):e0300318. doi: 10.1371/journal.pone.0300318 (PMC10986926; doi:10.1371/journal.pone.0300318)
Supplement: Attachment — Submitted filename: Response to Reviewers.docx [file pone.0300318.s004.docx]

**RESPONSE TO REVIEWERS**

**Manuscript ID** PONE-D-23-29198 “Development and Evaluation of the ARM Algorithm: A Novel Approach to Quantify Musculoskeletal Disorder Risk Factors in Manual Wheelchair Users in the Real World”

Key:

**Reviewer comment**

Response

*Edited Text*

Edits in the revised manuscript text are underlined and highlighted.

-------------------------------------------------------------------------------------------------

We thank the academic editor and reviewers for their thorough assessment of our manuscript and for their valuable feedback that have allowed us to improve the clarity and quality of the manuscript.

**If applicable, we recommend that you deposit your laboratory protocols in protocols.io to enhance the reproducibility of your results. Protocols.io assigns your protocol its own identifier (DOI) so that it can be cited independently in the future.**

The information for new method regarding algorithm development and protocols for data collection, processing, and analysis are included in detail in the methods section of the manuscript. For the methods and protocols for free-living environment data collection and calculation of humeral elevation from IMU data, that are well established, articles where these protocols are described in detail are cited, and sufficient information is included in the methods section of the manuscript to be to be understood independent of these references.

**Journal Requirements:**

**When submitting your revision, we need you to address these additional requirements**.

1. **Please ensure that your manuscript meets PLOS ONE's style requirements, including those for file naming.**

Title page has been edited and the manuscript formatting has been updated.

2. **Please note that PLOS ONE has specific guidelines on code sharing for submissions in which author-generated code underpins the findings in the manuscript. In these cases, all author-generated code must be made available without restrictions upon publication of the work.**

A pseudocode (Figure 1) describing the ARM algorithm, which is the main product of this study, is included in the methods section. Additionally, the ARM algorithm is described in detail in the Algorithm Development part of the methods section.

3. **We note that the grant information you provided in the ‘Funding Information’ and ‘Financial Disclosure’ sections do not match.**

**When you resubmit, please ensure that you provide the correct grant numbers for the awards you received for your study in the ‘Funding Information’ section.**

**4. Thank you for stating the following financial disclosure: 
 "This publication was made possible by funding from the National Institutes of Health (grant no. R01HD84423)."**

**Please state what role the funders took in the study.  If the funders had no role, please state: ""****The funders had no role in study design, data collection and analysis, decision to publish, or preparation of the manuscript."" 
If this statement is not correct you must amend it as needed. 
Please include this amended Role of Funder statement in your cover letter; we will change the online submission form on your behalf.**

The funders had no role in the study. We updated the Funding statement and financial disclosure and now reads:

Lines 399-400: This publication was made possible by funding from the National Institutes of Health (NIH; grant no. R01HD84423). *NIH had no role in study design, data collection and analysis, decision to publish, or preparation of the manuscript.*

**5. In your Data Availability statement, you have not specified where the minimal data set underlying the results described in your manuscript can be found. PLOS defines a study's minimal data set as the underlying data used to reach the conclusions drawn in the manuscript and any additional data required to replicate the reported study findings in their entirety. All PLOS journals require that the minimal data set be made fully available.**

**Upon re-submitting your revised manuscript, please upload your study’s minimal underlying data set as either Supporting Information files or to a stable, public repository and include the relevant URLs, DOIs, or accession numbers within your revised cover letter.**

A minimal dataset underlying the results for the free-living environment data collection for the dominant and non-dominant sides has been uploaded as a supporting information file (Free Living Environment Data.xlsx). Additionally, we have uploaded Appendix A and Appendix B, including participant characteristics in free-living environment data collection and a pictorial representation of the experimental set-up for community data collection as supporting information files.

**Additional Editor Comments:**
**All the reviewers have provided positive feedback on a study that monitors arm use in manual wheelchair users to assess musculoskeletal risks. As per their suggestions and my carefully reading, I recommend visual enhancements, referencing earlier work, and improved image quality. There are some concerns about result validity, suggesting the inclusion of more experts, clarifications about threshold choices, and additional graphics. Additionally, more clarity on accuracy in arm elevation measurement and socio-demographic information about participants should be highlighted. The authors are suggsted providing demographic information about the study sample and converting Figure 2 into a table.  The authors are finally suggested to address all the concerns raised by the reviwers, especially third reviewer to enhance the study's quality, validity, and presentation.** 

We appreciate the academic editor and reviewers for their thorough assessment of our manuscript and their valuable feedback. All the provided comments have been carefully addressed, and the manuscript has been revised in accordance with the suggestions made by the reviewers. We are confident that these revisions have significantly enhanced the clarity and overall quality of the manuscript.

**Reviewers' comments:**

**Comments to the Author**

We thank the reviewers for their thorough assessment of our manuscript. The manuscript has been revised according to the reviewers’ comments and suggestions.

**Reviewer #1:** **This study addresses a critical concern, the musculoskeletal risks faced by manual wheelchair users with spinal cord injuries. It offers a commendable approach, quantifying these risks in real-world settings using IMUs to monitor arm use during daily activities. The ARM algorithm, guided by ergonomics, promises crucial insights into repetitive arm motion. This research is a valuable contribution, with potential implications for the development of preventive programs to safeguard the shoulder health of manual wheelchair users.

However, while the research presents an innovative approach and a valuable contribution to the field, there are several critical points that need attention:**

We thank the reviewers for their thorough assessment of our manuscript. The manuscript has been revised according to the reviewers’ comments and suggestions.

**1. A pictorial representation or a real environment image of the experimental setup in the methodology section would significantly enhance the clarity and understanding of the study. Relying solely on textual descriptions can make the manuscript less accessible to readers. I strongly recommend the inclusion of such visuals in the sub-section “Participants and Data Collection” line 91.**

We have included a pictorial representation of the experimental protocol for community data collection (manual wheelchair propulsion and car transfer) as a supporting information file (Appendix B).

**Figure 1. Experimental Set-up.** Examples of experimental set-ups for community data collection (a & b): a) wheelchair propulsion in the community and b) car-to-wheelchair transfer with IMU sensors on upper arms.

**2. It would be beneficial to incorporate references to relevant earlier work by other researchers in the introduction section before line 80. Highlight the gaps in their research that make this study more impactful. This context can strengthen the paper's academic contribution.**

We appreciate the reviewer’s comment. To address this suggestion, we have incorporated references relevant to earlier work in ergonomics and revised the introduction section.

*Edited Text:* *Various tools have been devised and validated to screen repetitive tasks involving the upper extremities, aiming to assess the risks of work-related musculoskeletal disorders (15-17). Additionally, methods employing wearable sensors have been developed to objectively measure repetitive upper arm motions (18-20), facilitating the evaluation of physical exposure and MSK risks in the workplace. In contrast to ergonomic studies that focus on structured and known tasks, our study with MWC users analyzes unsupervised and unstructured free-living daily activities, providing a better reflection of natural habitual behavior. Consequently, the application of ergonomic definitions of repetition and quantitative methods directly to data from daily life is challenging, necessitating alternative metrics that offer a valid interpretation of arm use in a free-living environment.* *(Lines 70-80)*

*15. Hignett S, McAtamney L. Rapid entire body assessment (REBA). Applied ergonomics. 2000;31(2):201-5.*

*16. Lind C, Rose L. Shifting to proactive risk management: Risk communication using the RAMP tool. Agronomy Research. 2016;14(2):513-24.*

*17. McAtamney L, Corlett EN. RULA: a survey method for the investigation of work-related upper limb disorders. Applied ergonomics. 1993;24(2):91-9.*

*18. Lind CM, Abtahi F, Forsman M. Wearable Motion Capture Devices for the Prevention of Work-Related Musculoskeletal Disorders in Ergonomics—An Overview of Current Applications, Challenges, and Future Opportunities. Sensors. 2023;23(9):4259.*

*19. Peppoloni L, Filippeschi A, Ruffaldi E, Avizzano CA. A novel wearable system for the online assessment of risk for biomechanical load in repetitive efforts. International Journal of Industrial Ergonomics. 2016;52:1-11.*

*20.Thamsuwan O, Galvin K, Tchong-French M, Aulck L, Boyle LN, Ching RP, et al. Comparisons of physical exposure between workers harvesting apples on mobile orchard platforms and ladders, part 2: Repetitive upper arm motions. Applied ergonomics. 2020;89:103192.*

**3. The quality of the figures provided in the manuscript is suboptimal and appears to be blurry (Figures 1 and 3) I strongly suggest improving the image quality to ensure that they effectively convey the intended information.**

We have updated Figure 1 and Figure 3 (now referred to as Figure 2) with higher quality.

**4. A thorough proofreading and editing process is needed to address numerous grammatical, punctuation, and sentence structure errors in the manuscript. Enhancing the overall clarity and readability of the paper is essential.**

We have edited and proofread the manuscript, resulting in a notable improvement in its clarity.

**5. Although the inclusion and exclsuion criteria are described in [12], the authors are strongly suggested to briefly mention the same in the present work to improve the readability of the manuscript. Moreover, the level of SCI for MWC should be clearly highlighted.**

We appreciate the reviewer’s comment. To address this suggestion, we added a brief description of the inclusion and exclusion criteria to the methods section. The information regarding the level of SCI has been included in Table 2.

*Edited Text:* *Inclusion criteria included functional upper extremity range of motion, defined as active shoulder flexion and abduction of at least 150° and the ability of the participant to touch the opposite shoulder, the back of his/her neck and his/her low back. Participants were excluded if they self-reported a previous diagnosis of rotator cuff tendon tear. Additionally, MWC users were excluded if they had health complications that would inhibit their ability to participate in the larger longitudinal study such as stage IV pressure injuries, extensive comorbidities, or severe pain.* *(Lines 97-103)*

**6. How four different movement thresholds are selected? Then in lines 153-555, why number MWC propulsion cycles was evaluated with only three different movement thresholds? The authors have just dumped several pieces of information without adequate explanations. They are suggested to recheck the complete manuscript for such tangential information.**

As mentioned in the introduction section, the ARM algorithm development was inspired by ergonomic studies, particularly the study conducted by Thamsuwan et al. (2020). They introduced an approach to characterize the repetitive motions of the upper arms based on direct measurements using accelerometers. In this study, repetition rates were derived from upper arm inclination data. One cycle of motion was defined as the difference in angles between successive local maxima and minima when greater than a movement threshold. Various thresholds (ranging from 5° to 30° in increments of 5°) were used to calculate upper arm repetition, which was then compared to the repetition results from video-based observations. The study reported that as the threshold for the change in the upper arm angle increased from 5° to 30°, the calculated repetition rates decreased. The differences between the calculated upper arm repetition based on accelerometer data and video-based observations were statistically significant when the threshold ranged from 15° to 30°. In the present study, we assessed the feasibility of the ARM algorithm by employing comparable thresholds (ranging from 5° to 20° in 5° increments) for quantifying active bouts during repetitive arm motion in MWC users with SCI.

To assess the ARM algorithm's suitability in characterizing arm use during repetitive activities, we estimated the number of manual wheelchair (MWC) propulsion stroke cycles using the ARM algorithm with three distinct movement thresholds (5°, 10°, 15°). This estimation was then compared to the actual number of stroke cycles obtained from video data. We deliberately omitted the use of a 20° threshold due to our observations, indicating a limited range of motion in the upper arm during MWC propulsion, especially on hard, level surfaces. For instance, applying the ARM algorithm with a 20° threshold resulted in a 70% underestimation of the number of propulsion cycles in one participant.

We have incorporated this information into both the Methods and Discussion sections to provide a clearer explanation of our criteria for selecting the four distinct movement thresholds and to justify our decision to use only three of these thresholds for estimating the number of propulsion cycles. Furthermore, we have delved into the importance of conducting a validation study with a larger sample size, emphasizing the utilization of optimization algorithms for determining the optimal threshold. This approach is crucial for ensuring accurate analysis of musculoskeletal disorder risk factors in MWC users with SCI, particularly in the context of repetitive arm motion.

*Edited Text: The rated video data (propulsion activity and non-propulsion activity) were used as criterion standard data to evaluate the accuracy of the estimated active and resting times based on four different movement thresholds (5°, 10°, 15°, 20°). We selected these movement thresholds based on ergonomic literature, particularly the study conducted by Thamsuwan et al.(20), which used accelerometers to characterize upper arm repetitive motions. Their study revealed that increasing the threshold (5° to 30°) led to a decrease in calculated repetition rates, with significant differences observed at 15° to 30°.(Lines 175-182)*

*To evaluate the applicability of this approach for characterization of arm use during active bouts, the number of MWC propulsion stroke cycles, as an exemplar of repetitive arm activities, was estimated using the ARM algorithm with three different movement thresholds (5°, 10°, 15°) and compared with the actual number of stroke cycles from video data (propulsion activity). We deliberately omitted the use of a 20° threshold due to our observations, indicating a limited range of motion in the upper arm during MWC propulsion, especially on hard, level surfaces. (Lines 183-188)*

*This study was an essential step towards our long-term research goal to develop a proactive approach for early mitigation of shoulder pathologies in MWC users with SCI and help this population decelerate shoulder soft tissue “aging” while maintaining a full and active lifestyle. However, conducting a validation study with a larger sample size and utilizing optimization algorithms to determine the optimal movement threshold is crucial for ensuring an accurate analysis of musculoskeletal disorder risk factors in MWC users with SCI in the context of repetitive arm motion. (Lines 371-377)*

**7. The authors are suggested to highlight the critical insights and plausible implications of the observations for In-home and Free Living Environment Data. At present authors have just mentioned the Figures and Tables without any explanations. For example: in Lines 209-211, 'Table 5 demonstrates how the ARM algorithm....task', it is still unclear how?. Similarly, in Lines 220-224, if during long active bouts, the total time and other factors were significantly higher for the dominant side, what's the significance in the context of the MWC users?**

We appreciate the reviewer’s comments. To address these comments, we have updated the results section and highlighted the critical insights of the observation for both in-home and free-living environment data in the discussion section (Lines 286-340). Additionally, we have removed the terminology "long active bout" from the manuscript and discussed the necessity of further research to establish a clinically relevant threshold for the length of active bouts to be included in the analysis in the discussion section.

*Edited Text:*

*Table 5 indicates longer active time and shorter resting time for the dominant arm compared to the non-dominant arm during in-home activities for both participants. Table 6 indicates the counts of movement cycles, along with the median and peak humeral elevation angles during daily activities. These measurements signify repetition and awkward postures, recognized as key extrinsic risk factors for musculoskeletal disorders in the ergonomics literature. (Lines 242-247).*

*These findings suggest that focusing on active bouts exceeding 10 seconds in duration may yield more meaningful insights compared to analyzing all active bouts indiscriminately. This underscores the need for future studies to establish a clinically relevant threshold for the inclusion of active bouts in the analysis.* (Lines 337-340).

**8. Finally, the authors are suggested to compare the results of the present work (ARM Algorithm) with state-of-the-art methods, either qualitatively or quantitatively in the Discussion section. Moreover, the limitations of the present work should be explicitly highlighted at the end of the Discussion section.**

We appreciate the reviewer's comment. We have added a paragraph comparing the results of the present work with state-of-the-art methods in ergonomic research. The limitations of this study are highlighted in the last two paragraphs of the discussion section.

*Edited Text:*

*The ARM algorithm, proposed in this study, was informed by ergonomic research, notably the work conducted by Thamsuwan and colleagues(20). Their successful development of a method, utilizing accelerometers, to assess repetitive arm motions in apple-harvesting workers served as a foundational reference. The fundamental definition of movement cycles within the ARM algorithm aligns with the repetition cycles identified in Thamsuwan’s study, and both studies found comparable thresholds to have the highest accuracy in calculating repetition rates. The utilization of IMUs in the current study provided a better assessment of arm use in MWC users in the free-living environment compared to using only accelerometers, as humeral elevation can be calculated more accurately using both linear acceleration and angular velocity comparted to only linear accelerometer readings. The incorporation of IMUs in the current study offered a more comprehensive assessment of arm usage among MWC users in a free-living environment compared to relying solely on accelerometers. Additionally, the ARM algorithm was employed to evaluate bouts of arm use within the context of repetitive arm movements, providing a holistic analysis of the physical risk factors associated with shoulder musculoskeletal disorders in MWC users. (Lines 341-358)*

**Reviewer #2:**

1. **Is the manuscript technically sound, and do the data support the conclusions?**

**The main issue with the validity of the results is that the ground truth is provided by and dependent on only one expert. Would the addition of another expert result on a different threshold value choice? If that so, would then the (already minimal) differences/size effects become insignificant? The lack of an automated way to calculate the ground truth timings would require providing more experts assessments for consensus.**

We appreciate the reviewer for this comment. We agree that having more than one rater would increase the reliability of the methods for rating the video data. However, we do not expect a different threshold value choice with adding more raters. As explained in the methods section, the rater was instructed to classify the video data as resting, propulsion activity, or non-propulsion activity (excluding small fidgeting movements) and to determine the starting and ending times for each activity. The rater did not rate arm movements or the magnitude of the arm elevation. Later, the rated data were used to evaluate the accuracy of the estimated active and resting times based on four different movement thresholds (5°, 10°, 15°, 20°) using the ARM algorithm. Additionally, our team previously determined that the intraclass correlation coefficients for inter-rater reliability were greater than 0.95 for static orientations and movement classifications by two raters (<https://www.sciencedirect.com/science/article/pii/S135045331300146X?via%3Dihub> )

We since only use one rater for video analyses as in Goodwin et al.

(<https://www.mdpi.com/1424-8220/21/4/1236> )

and Fortune et al.

(<https://www.sciencedirect.com/science/article/pii/S1050641118305157> )

To address this comment, we updated the methods section and mentioned the lack of inter-rater and intra-rater reliability analysis, in addition to the small sample size, as limitations of this study that should be addressed in future studies.

*Edited Text:*

*Methods*

*One researcher (OJ, with greater than 9 years of movement analysis experience) rated the video data as resting, propulsion activity, or non-propulsion activity by determining the starting and ending times for each activity (excluding small fidgeting movements). (Lines 173-175)*

Discussion

*Furthermore, the study's limitations, such as having only one expert for video data rating and the absence of intra-rater and inter-rater reliability analyses, should be acknowledged and addressed in future studies.* *(Lines 378-380)*

**Other minor issues are:
- What was the rationale for choosing 10 seconds as a long active bout when the threshold was 7 seconds?**

In this study if intervals of 'no activity' less than 7 seconds between two identified active segments (movement cycles) were less than 7 seconds then the segments and the intervals between them were merged together and considered to make up one active bout. Thus, the active bout could be much shorter than 7 seconds (for example 2 seconds) or much longer. Therefore, in addition to analyzing all active bouts, we also analyzed the active bouts which were longer than 10 second. We did not have any objective criterion for choosing 10 seconds, but the results indicated the necessity of further research to establish a clinically relevant threshold for the length of active bouts to be included in the analysis in the discussion section.

To address this comment, we have removed the terminology "long active bout" from the manuscript and discussed the necessity of further research to establish a clinically relevant threshold for the length of active bouts to be included in the analysis in the discussion section.

*Edited Text:*

*Active and Resting Bouts:* *Next the intervals (time) of no activity between the consecutive movement cycles were calculated. An activity bout contains one or more movement cycles that may be separated by no activity intervals up to 7 seconds. A no activity interval of greater than 7 seconds marks the end of an activity bout (22), (Fig 1, b).* *Seven seconds was chosen in a study specific to wheelchair propulsion by Tolerico et al. as the amount of time with no wheelchair activity in which the participant was considered not-active (22), and that “resting” time frame was also used in this study. The intervals of no activity greater than 7 seconds were considered resting bouts (Fig 1, c). Finally, the number of movement cycles per activity bout and the kinematic and temporal features of each cycle and each activity bout were calculated (Table 1). Metrics were calculated for all active bouts as well as active bouts that were longer than 10 seconds. (Lines 136-146).*

*These findings suggest that focusing on active bouts exceeding 10 seconds in duration may yield more meaningful insights compared to analyzing all active bouts indiscriminately. This underscores the need for future studies to establish a clinically relevant threshold for the inclusion of active bouts in the analysis.* *(Lines 337-340).*

- **While the effect of drift was mentioned in the Discussion section the manuscript lacks a deeper explanation of the algorithm for humeral elevation angle calculation. Hence, there is uncertainty about the accuracy of the IMU recordings for the long periods in the free-living dataset.**

In this study, orientation estimates are derived by combining data from both acceleration and angular velocity within a sensor fusion framework, resulting in superior orientation estimates. The basic process is illustrated well by the following figure (Figure 4) and caption from Ojeda et al. (2017):

In a common sensor fusion framework, angular velocity is integrated to provide an estimate of orientation. It is understood that the estimated orientation will ‘drift’ away from the true orientation. To correct this drift, measurements from the accelerometer during times of low dynamics (measured acceleration close to 1g) can be used to correct the orientation by providing a ‘measurement’ of the vertical direction (an accelerometer at rest acts as an inclinometer). Corrections using measurements from the accelerometer help correct the ‘tilt’ or pitch and roll of the orientation estimate. If the inertial sensor contains a magnetometer and the sensor is used in areas where the magnetic field is uniform or well known, measurements of the direction of magnetic north can be used to correct the yaw or heading direction of the orientation (Sabatini, 2006). Therefore, in our study, we trust estimates of orientation relative to gravity (humeral elevation) but cannot trust estimates of yaw or heading (required to estimate the full humeral-thoracic angle) because the magnetic fields experienced by our participants are unknown.

Ojeda, L.V., Zaferiou, A.M., Cain, S.M., Vitali, R.V., Davidson, S.P., Stirling, L.A., Perkins, N.C., 2017. Estimating stair running performance using inertial sensors. Sensors (Basel) 17, 2647.

Sabatini, A.M., 2006. Quaternion-based extended Kalman filter for determining orientation by inertial and magnetic sensing. IEEE Transactions on Biomedical Engineering 53, 1346-1356.

To address this comment, we have incorporated a summary of our methods for calculating humeral elevation angles and referenced publications from our group that provide a comprehensive description of these methods Furthermore, we have made updates to the discussion section.

Edited Text:

Methods

*Participants were asked to wear three IMUs (Opal or Emerald, APDM Inc., Portland, Oregon), one on each upper arm and one on their chest. Additionally, participants were asked to perform a functional calibration at the beginning of data collection (8). Our established method was used to calculate the humeral elevation angle (8, 12).* *In summary, a sensor fusion method (Kalman filter) was employed to estimate inertial measurement unit (IMU) orientation from linear acceleration and angular velocity (21). Custom MATLAB code (MathWorks, Natick, MA) was developed to calculate orientations of anatomical axes relative to IMU-fixed reference frames using data collected during each participant’s functional calibration postures and movements (8). The orientation of the upper arm in an inertial (world) reference frame was then estimated using the orientation of the IMU and the orientation of the anatomical axes relative to the IMU-fixed reference frame. The humeral elevation angle was defined as the angle between the long axis of the body segment and the vertical. These angles depend solely on the estimated direction of gravity relative to the body segment, making them drift-free metrics for quantifying body segment motions (8). The calculated humeral elevation angles range between 0–180°, with 0° indicating the arm was down and perfectly aligned with gravity, and 180° indicating the arm was raised overhead and aligned with gravity. These methods have previously been validated in unpublished data involving five individuals with SCI who completed 10 reaching tasks. In comparison to the gold standard (electromagnetic system), the absolute error and percentage of error for the range of motion were found to be -0.06±1.12° and -1.44±1.28%, respectively. Similarly, for the maximum elevation achieved during each reach, the absolute error and percentage of error were 2.59±2.47° and 2.04±2.47%, respectively. (Lines 106-126)*

Discussion

*The small sample size in this study restricted the extent to which these findings can be generalized. Additionally, we can only trust estimates of body segment orientation relative to vertical. Because data collections took place in free-living environments with non-uniform magnetic fields, we did not use magnetometer measurements in our sensor fusion approach for calculating orientations. Therefore, orientation calculations relative to magnetic north are not reliable (i.e., the yaw or heading angle is contaminated by uncorrected integration drift) and are not suitable for accurate calculation of humerothoracic elevation angles and elevation planes relative to the thorax (8, 25). Therefore, shoulder flexion or abduction were both interpreted as humeral elevation and are indistinguishable. (Lines 356-364)*

**- More graphics (e.g. boxplots, violin plots) could improve understanding of the data and their distribution. Tables could be used as supplementary information.**

We utilized boxplots with individual data points for the free-living environment data (Figure 2). However, due to the small sample size in the community and in-home data, we considered reporting mean (SD) and median (IQR) for these datasets. Additionally, a minimal dataset underlying the results for the free-living environment data collection for the dominant and non-dominant sides has been uploaded as a supporting information file (Free Living Environment Data.xlsx).

**- There seem to be outliers driving the statistical significance in Fig. 3f and Fig. 3g.**

There were no outliers in the free-living environment (n=16) dataset. Additionally, the statistical analysis method (Wilcoxon Signed-rank) used in this study relies on the ranking of data rather than their specific values. The results for active bout duration (Fig. 3f) and the number of cycles per active bout (Fig. 3g) during active bouts longer than 10 seconds indicated that, for 12 and 13 participants (out of 16), respectively, the average duration of active bouts and the average number of cycles per active bout were lower for their non-dominant side than their dominant side.


**Is the manuscript presented in an intelligible fashion and written in standard English?**

**More concise writing would improve the manuscript readability.**

**For instance, the text in lines 113-117 was unclear as the resting and active bouts share the same threshold value (one under and another above threshold), but they were referred to as different thresholds, which could be confusing/repetitive.**

We used the same movement threshold for identifying arm elevation and arm lowering in this study. An active bout was defined as consecutive movement cycles with intervals of less than 7 seconds, and intervals greater than 7 seconds were considered resting bouts. We updated the methods section to improve clarity.

Edited Text:

*Repetition: To define repetition in the free-living environment, first the basic pattern of one arm movement cycle was identified. One movement cycle was defined as an arm elevation greater than a movement threshold (for example 10°), followed by an arm lowering greater than the movement threshold. Movements with smaller ranges of motion than the movement threshold that occurred between successive elevation and lowering events were termed ‘idle’. Idle periods were treated as a part of the movement cycle (Fig 1, a). A range of thresholds were investigated to determine the optimal movement threshold.*

*Active and Resting Bouts: Next the intervals (time) of no activity between the consecutive movement cycles were calculated. An activity bout contains one or more movement cycles that may be separated by no activity intervals up to 7 seconds. A no activity interval of greater than 7 seconds marks the end of an activity bout (22), (Fig 1, b). Seven seconds was chosen in a study specific to wheelchair propulsion by Tolerico et al. as the amount of time with no wheelchair activity in which the participant was considered not-active (22), and that “resting” time frame was also used in this study. The intervals of no activity greater than 7 seconds were considered resting bouts (Fig 1, c). Finally, the number of movement cycles per activity bout and the kinematic and temporal features of each cycle and each activity bout were calculated (Table 1). Metrics were calculated for all active bouts as well as active bouts that were longer than 10 seconds. (Lines 129-146)*

**Line 207 should say “respectivelly” instead of “respectfully**”.

Manuscript has been edited and proofread.

**Reviewer #3: Relevant topic, nicely written manuscript.

However, some topics need to be addressed/elaborated.

Intro: Clearly written, no comments**

**Methods:**
**- it would be good to mention the number of participants included in each portion of the study in the methods section.**

The number of participants have included in table 2 (Description of the data sets) and added in the text.

**- When using a threshold for arm elevation, the accuracy of determining arm elevation angles should be addressed.**

To address this comment, we have incorporated a summary of our methods for calculating humeral elevation angles and referenced publications from our group that provide a comprehensive description of these methods.

Edited Text:

Methods

*Participants were asked to wear three IMUs (Opal or Emerald, APDM Inc., Portland, Oregon), one on each upper arm and one on their chest. Additionally, participants were asked to perform a functional calibration at the beginning of data collection (8). Our established method was used to calculate the humeral elevation angle (8, 12). In summary, a sensor fusion method (Kalman filter) was employed to estimate inertial measurement unit (IMU) orientation from linear acceleration and angular velocity (21). Custom MATLAB code (MathWorks, Natick, MA) was developed to calculate orientations of anatomical axes relative to IMU-fixed reference frames using data collected during each participant’s functional calibration postures and movements (8). The orientation of the upper arm in an inertial (world) reference frame was then estimated using the orientation of the IMU and the orientation of the anatomical axes relative to the IMU-fixed reference frame. The humeral elevation angle was defined as the angle between the long axis of the body segment and the vertical. These angles depend solely on the estimated direction of gravity relative to the body segment, making them drift-free metrics for quantifying body segment motions (8). The calculated humeral elevation angles range between 0–180°, with 0° indicating the arm was down and perfectly aligned with gravity, and 180° indicating the arm was raised overhead and aligned with gravity. These methods have previously been validated in unpublished data involving five individuals with SCI who completed 10 reaching tasks. In comparison to the gold standard (electromagnetic system), the absolute error and percentage of error for the range of motion were found to be -0.06±1.12° and -1.44±1.28%, respectively. Similarly, for the maximum elevation achieved during each reach, the absolute error and percentage of error were 2.59±2.47° and 2.04±2.47%, respectively. (Lines 106-126)*

**Lines 152-156
- wc-propulsion was used as an exemplar of repetitive movements. Were any other movement activities (e.g. above-shoulder height arm elevation) also included? As those movements were mentioned to be risk factors for musculoskeletal disorders in the introduction!**

As listed in Table 2, community data collection included MWC-based activities, with various episodes of above-shoulder height arm elevation observed during tasks such as car transfers and reaching. Moreover, during in-home data collection, particularly in meal preparation, numerous instances of above-shoulder height arm elevation were noted. By identifying bouts of activity in the context of repetitive upper arm movements, we were able to assess both arm elevation and repetitive arm movements—both recognized as risk factors for shoulder musculoskeletal disorders. This approach holds potential for classifying activities and indirectly providing insights into loading, another significant risk factor for shoulder musculoskeletal disorders. *(discussion section)*

- **Could the observer rate the amount of movement of above shoulder movements (e.g. Range of motion) with sufficient accuracy from the 2 dimensional video, to compare with output of the ARM algorithm?? Especially those movements around the thresholds used (5°, 10° and 15°)?**

Similar to our response to a similar comment by the second reviewer, we’ve included text in the methods section to explain that the observer (rater) in this study was instructed to classify the video data as resting, propulsion activity, or non-propulsion activity (excluding small fidgeting movements) and to determine the starting and ending times for each activity. The rater did not assess arm movements or the magnitude of arm elevation. Later, the rated data were used to evaluate the accuracy of the estimated active and resting times based on four different movement thresholds (5°, 10°, 15°, 20°) using the ARM algorithm. Therefore, the rater did not directly evaluate the movement of the arm but rather assessed wheelchair-based activities in the community.

To provide better clarification, we updated the methods section and acknowledged the absence of inter-rater and intra-rater reliability analyses, along with the small sample size, as limitations of this study. These aspects should be addressed in future studies.

*Edited Text:*

*Methods*

*One researcher (OJ, with greater than 9 years of movement analysis experience) rated the video data as resting, propulsion activity, or non-propulsion activity by determining the starting and ending times for each activity (excluding small fidgeting movements). (Lines 173-175)*

Discussion

*Furthermore, the study's limitations, such as having only one expert for video data rating and the absence of intra-rater and inter-rater reliability analyses, should be acknowledged and addressed in future studies.* *(Lines 378-380)*

**- In line 152 four thresholds are mentioned, in line 155 only three. Which thresholds were actually used for this comparison?**

Please see the response to a similar comment by the first reviewer (comment # 6)

**Lines 158 – 167:**
**- It is completely valid to ask the participants to do only portions of tasks for this specific reason of comparison. Question is how much data should be collected to have a fair comparison. Do you have any information/arguments for such?**

In this study, for free-living data collection, only 1 or 2 days of data were collected for participants due to participant availability. We acknowledge that this may not be a reliable representation of their arm use throughout a week.

Previously, our reliability analysis indicated that at least five days of data are needed from the MWC users to achieve a reliable representation of their overall daily arm use throughout a week, including both weekdays and weekends. However, only four days of data might be needed to obtain a reliable representation of arm use during weekdays only for MWC users. The difference between the weekday and weekend analysis and the only weekday analysis is primarily due to variations in arm use intensity levels during weekdays versus weekends.

- Goodwin BM, Jahanian O, Van Straaten MG, Fortune E, Madansingh SI, Cloud-Biebl BA, et al. Application and Reliability of Accelerometer-Based Arm Use Intensities in the Free-Living Environment for Manual Wheelchair Users and Able-Bodied Individuals. Sensors. 2021;21(4):1236.

Schneider et al. also investigated the reliability of wheelchair-based metrics from wrist-worn accelerometry data for individuals with SCI who use MWCs. The metrics included measures of physical activity intensity, wheeling quantity, and movement quality from data collected during in- and out-patient settings. Their results suggest that four days of data are needed for good reliability of movement quality, and three days are required for most other metrics in the out-patient setting. They found no difference between weekdays and weekends in wheelchair distance traveled.

- Schneider S, Popp WL, Brogioli M, Albisser U, Demkó L, Debecker I, et al. Reliability of wearable-sensor-derived measures of physical activity in wheelchair-dependent spinal cord injured patients. Frontiers in neurology. 2018;9:1039.

Additionally, a pilot study by our team (unpublished) with 6 MWC users indicated that up to 5 days of data might be needed to achieve a reliable representation (reliability > 0.75) of risk and recovery metrics of arm use throughout a week.

This limitation has been mentioned in the discussion section) and will be addressed in our future studies by including more days of IMU data collection.

- **Furthermore, data from two participants seems rather marginal for such comparison and validation. (and yes, I do know video annotation is time consuming, but that shouldn't count as argument not to include more participants, it is about the validity of your method) Please elaborate here.**

We acknowledge that small sample size is a limitation in this study that restrict the extent to which the findings of this study can be interpreted and generalized. We have mentioned this limitation in the discussion section (Lines 356-366) and have elaborated the necessity of conducting a validation study with a larger sample size to determine the optimal movement threshold is crucial for ensuring an accurate analysis of musculoskeletal disorder risk factors in MWC users with SCI in the context of repetitive arm motion (Lines 356-366).

**Results:
The results section could benefit a short description of the study sample, in terms of socio demographics and lesion severity, especially for the unsupervised Community Environment data.**

We have uploaded Appendix A, including participant characteristics in free-living environment data collection as supporting information files.

Table 1. Participant characteristics in free-living environment data collection (N= 16)

| **Sex, N(%)** | |
| --- | --- |
| Female | 2 (12%) |
| Male | 14 (88%) |
| **Age, years** | |
| Mean (SD) | 41 (12) |
| Median (IQR) | 37 (31, 55) |
| **Duration of wheelchair use, years** | |
| Mean (SD) | 11 (12) |
| Median (IQR) | 6 (4, 12) |
| **Injury level, N(%)** | |
| C6-C8 | 3 (19%) |
| T1-T8 | 8 (50%) |
| T9-L1 | 5 (31%) |
| **AIS Score, N(%)** | |
| A | 9 (56%) |
| B | 3 (19%) |
| Unknown | 4 (25%) |
| **Self-reported Completeness of SCI, N(%)** | |
| Complete | 8 (50%) |
| Incomplete | 8 (50%) |

**Discussion:
The discussion section mostly discusses results obtained with the ARM method. Although relevant, some more attention should be paid to the strengths and weakness of the ARM method in getting to these results: how valid is the method? When that question is answered, then and only then the results of the application thereof are of interest. Strength and weakness should be addressed in a bit more detail, e.g. with respect to validity on above shoulder arm elevation. eventual a limitation section should be added to the manuscript.**

We appreciate the reviewer for their suggestions. In response to these comments, we have revised the discussion section, addressing the limitations and outlining potential future directions for this study in the last two paragraphs of the discussion section.

**Figures are clear, especially figure 1, explaining the details in the definition of a movement cycle.
Figure 2 should be converted to a compact table, in my modest opinion :-)**

We converted Figure 2 to a compact table, now labeled as Table 4.

**Table 4. Estimated number of stroke cycles when using different movement thresholds (5-15 degrees).** Negative errors indicate underestimation and positive errors indicate overestimation.

|  | Actual | 5 Deg | 10 Deg | 15 Deg |
| --- | --- | --- | --- | --- |
| **Number of Stroke cycles** | 1116 | 1204 | 1091 | 780 |
| **Error** | N/A | 8% | -2% | -30% |
